# Supplementary material for: Validation of a French version of the Breakthrough Pain Assessment Tool in cancer patients: Factorial structure, reliability and responsiveness
Source: PLoS One. 2023 Jul 10;18(7):e0286947. doi: 10.1371/journal.pone.0286947 (PMC10332612; doi:10.1371/journal.pone.0286947)
Supplement: S1 File — (DOCX) [file pone.0286947.s001.docx]

**Additional file 1:** French version of the Breakthrough Pain Assessment Tool (OFEA)

**OFEA**

**Les questions suivantes concernent vos accès douloureux paroxystiques de ces sept derniers jours.**

**Un accès douloureux paroxystique est une augmentation de courte durée de vos douleurs cancéreuses.**

1. **
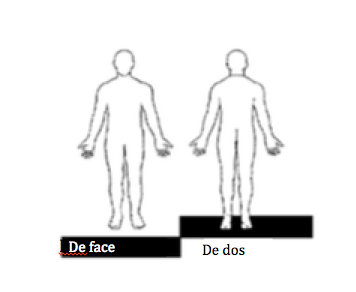
Où se situent vos accès douloureux paroxystiques ? Veuillez les situer sur le dessin par une croix (X)**
2. **À quelle fréquence présentez-vous des accès douloureux paroxystiques ? Veuillez entourer une réponse**

Moins d’une fois par jour ❒ 1-2 fois par jour ❒ 3-4 fois par jour ❒ Plus de 4 fois par jour❒

1. **Existe-t-il un élément qui déclenche vos accès douloureux paroxystiques ?**

**Si oui, veuillez noter lequel** ………………………………………………………………………………………………………………………....

………………………………………………………………………………………………………………………………………………………………………...

1. **Existe-t-il un élément qui soulage vos accès douloureux paroxystiques (antalgiques ou autre) ?**

**Si oui, veuillez noter lequel** ……………………………………………………………………………………………………………………………..

…………………………………………………………………………………………………………………………………………………………………………..

1. **Combien de temps dure habituellement un accès douloureux paroxystique  ? Veuillez entourer une réponse**

<5 min❒ 5-15 min ❒ 15-30 min❒ 30-60 min❒ >60 min❒

**6- Quelle est l’intensité de votre pire accès douloureux paroxystique ? Veuillez entourer un chiffre**

0 1 2 3 4 5 6 7 8 9 10

Pire douleur imaginable

Aucune douleur

**7-Quelle est l’intensité d’un accès douloureux paroxystique habituel ? Veuillez entourer un chiffre**

0 1 2 3 4 5 6 7 8 9 10

Pire douleur imaginable

Aucune douleur

**Les questions suivantes concernent vos accès douloureux paroxystiques de ces sept derniers jours.**

**Un accès douloureux paroxystique est une augmentation de courte durée de vos douleurs cancéreuses.**

**8- À quel point les accès douloureux paroxystiques vous angoissent-ils ? Veuillez entourer un chiffre**

0 1 2 3 4 5 6 7 8 9 10

Pas du tout Beaucoup

1. **À quel point les accès douloureux paroxystiques vous empêchent-ils de vivre une vie normale ?**

**Veuillez entourer un chiffre**

0 1 2 3 4 5 6 7 8 9 10

Pas du tout Beaucoup

1. **Quels antalgiques prenez-vous contre les accès douloureux paroxystiques (si vous en prenez) ?**

**Veuillez noter le type et la dose d’antalgiques** : ………………………………………….........................................................…………

…………………………………………………………………………………………………………………………………………………………………………………………….

1. **À quel point l’antalgique que vous prenez habituellement est-il efficace contre vos accès douloureux paroxystiques ? Veuillez entourer un chiffre**

0 1 2 3 4 5 6 7 8 9 10

Complètement efficace

Pas du tout efficace

1. **Combien de temps faut-il pour que l’antalgique ait un effet significatif sur vos accès douloureux paroxystiques ? Veuillez entourer une réponse**

Aucun effet ❒ 0-10 min ❒ 10-20 min❒ 20-30 min❒ >30 min❒

1. **Avez-vous des effets secondaires dus aux antalgiques que vous prenez contre vos accès douloureux paroxystiques ? Si oui, veuillez noter le type d’effets secondaires** :……………………………………………………………………………

…………………………………………………………………………………………………………………………………………………………………………………..

1. **À quel point êtes-vous gêné par les effets secondaires des antalgiques que vous prenez contre vos accès douloureux paroxystiques ? Veuillez entourer un chiffre**

0 1 2 3 4 5 6 7 8 9 10

Pas du tout Beaucoup
