# Supplementary material for: Validation of a French version of the Breakthrough Pain Assessment Tool in cancer patients: Factorial structure, reliability and responsiveness
Source: PLoS One. 2023 Jul 10;18(7):e0286947. doi: 10.1371/journal.pone.0286947 (PMC10332612; doi:10.1371/journal.pone.0286947)
Supplement: S2 File — CFI: comparative fit index; TLI: Tucker-Lewis Index; RMSEA: root mean square error of approximation; CI: Confidence interval; SRMR: Standardized Root Mean Square Residual. (DOCX) [file pone.0286947.s002.docx]

**Additional file 2:** Indexes of fit for exploratory factorial analysis models with one to four dimensions estimated with data of n=130 patients

| Number of dimensions | CFI | TLI | RMSEA (90% IC)  p-value | SRMR |
| --- | --- | --- | --- | --- |
| 1 | 0.56 | 0.41 | 0.126 [0.095 – 0.157]  p<0.001 | 0.14 |
| 2 | 0.91 | 0.83 | 0.067 [0.000 – 0.111]  p=0.245 | 0.07 |
| 3 | 1.00 | 0.99 | 0.013 [0.000 – 0.091]  p=0.690 | 0.04 |
| 4 | 0.99 | 0.94 | 0.04 [0.000 – 0.126]  p=0.49 | 0.02 |

CFI: comparative fit index; TLI: Tucker-Lewis Index; RMSEA: root mean square error of approximation; CI: Confidence interval; SRMR: Standardized Root Mean Square Residual.
