# Supplementary material for: Validation of a French version of the Breakthrough Pain Assessment Tool in cancer patients: Factorial structure, reliability and responsiveness
Source: PLoS One. 2023 Jul 10;18(7):e0286947. doi: 10.1371/journal.pone.0286947 (PMC10332612; doi:10.1371/journal.pone.0286947)
Supplement: S3 File — (DOCX) [file pone.0286947.s003.docx]

**Additional file 3:** Items grouping after CF-EQUAMAX rotation: estimate of the factor loadings associated to each of the two dimensions of the French BAT estimated with data of n=130 patients

| French BAT Items | D1 | D2 |
| --- | --- | --- |
| How often do you get breakthrough pain? (n°2) | **0.282** | -0.058 |
| How long does a typical episode last? (n°5) | 0.194 | **0.432*** |
| How severe is the worst breakthrough pain? (n°6) | **0.723*** | -0.017 |
| How severe is a typical breakthrough pain? (n°7) | **0.746*** | -0.062 |
| How much does the breakthrough pain distress you? (n°8) | **0.245** | 0.187 |
| How much does the breakthrough pain stop you from living a normal life? (n°9) | **0.556*** | 0.196 |
| How effective is the painkiller for your breakthrough pain? (n°11) | -0.091 | **-0.452*** |
| How long does the breakthrough painkiller take to have a meaningful effect? (n°12) | -0.044 | **0.899*** |
| How much do the side effects from your breakthrough painkiller bother you? (n°14) | 0.020 | **0.479*** |

**Bold**: item reflecting more strongly the dimension; *: factor loading >0.400; D1: Dimension 1 of the French BAT; D2: Dimension 2 of the French BAT.
