# Supplementary material for: Validation of a French version of the Breakthrough Pain Assessment Tool in cancer patients: Factorial structure, reliability and responsiveness
Source: PLoS One. 2023 Jul 10;18(7):e0286947. doi: 10.1371/journal.pone.0286947 (PMC10332612; doi:10.1371/journal.pone.0286947)
Supplement: S4 File — The condition number is an index of good quality of numerical results (ratio of smallest to largest eigenvalue for the Information Matrix, good if >10−6) e.g. no problem as multicolinearity of items. (DOCX) [file pone.0286947.s004.docx]

**Additional file 4:** Table reporting the goodness-of-fit and model assumptions of the EFA models. The condition number is an index of good quality of numerical results (ratio of smallest to largest eigenvalue for the Information Matrix, good if >10^-6^) e.g. no problem as multicolinearity of items.

| Number of Dimensions of EFA Model | Number of Free Parameters | Chi-Square Test of Model Fit for the Baseline Model | RMSEA (90% IC); p-value | CFI | TLI | SRMR | Condition number |
| --- | --- | --- | --- | --- | --- | --- | --- |
| 1 | 15 | P<0.0001 | 0.126 [0.095 – 0.157] ; P<0.001 | 0.556 | 0.408 | 0.136 | >10^-6^ |
| 2 | 23 | P<0.0001 | 0.067 [0.000 – 0.111] ; P=0.245 | 0.911 | 0.831 | 0.067 | >10^-6^ |
| 3 | 30 | P<0.0001 | 0.013 [0.000 – 0.091] ; P=0.690 | 0.998 | 0.994 | 0.040 | <10^-6^ for CF-EQUAMAX |
| 4 | 36 | P<0.0001 | 0.04 [0.000 – 0.126] ; P=0.49 | 0.990 | 0.940 | 0.024 | >10^-6^ |
