# Supplementary material for: Validation of a French version of the Breakthrough Pain Assessment Tool in cancer patients: Factorial structure, reliability and responsiveness
Source: PLoS One. 2023 Jul 10;18(7):e0286947. doi: 10.1371/journal.pone.0286947 (PMC10332612; doi:10.1371/journal.pone.0286947)
Supplement: S5 File — (PDF) [file pone.0286947.s005.pdf]

N_SUJET	V1_DF_CALME	V1_DF_TTT	V1_ADP_CALME	V1_ADP_TTT	D_REMPLISSAGE	SEXE	D_NAISSANCE	CANCER	AUTRE	D_DIAGNOSTIC	STADE	TTT_CANCER	OMS	ETIO_DOUL	DOUL_LIEE	PATIENT_VU	V1O_ELTS_SOULAGE	LQL_SOULAGE	V1O_DUREE
N° Sujet	Avez-vous l'impression que votre patient soit bien calmé au niveau de sa douleur de fond	Pensez-vous devoir modifier le traitement de la douleur de fond de votre patient	Avez-vous l'impression que les ADP de votre patient soient bien calmées	Pensez-vous devoir modifier le traitement des ADP de votre patient	Date de remplissage Visite 1	Sexe	Date de naissance	Diagnostic du cancer	Autre diagnostic de cancer	Date de diagnostic	Stade du cancer	Type de traitement	STADE OMS	Etiologie de la douleur	La douleur est liée	Le patient est vu	Existe-t-il un élément qui soulage vos accès douloureux paroxystiques ? Si oui, veuillez noter lequel	Si oui, veuillez noter lequel	Combien de temps dure habituellement un accès douloureux paroxystique
1	OUI	NON	OUI	NON	31/03/2015	FEMME	15/02/1953	hématologique	999	14/06/2012	Recidive	Chimiothérapie	2	mixte	cancer	hospitalisation	OUI	toucher	>60min
2	OUI	OUI	OUI	NON	30/03/2015	HOMME	15/04/1945	urologique	999	01/06/2012	Métastatique	Chimiothérapie	2	nociceptive	cancer	consultation	OUI	antalgiques	>60min
3	NC	NC	NC	NC	02/04/2015	FEMME	15/04/1949	gastro-intestinal	999	01/09/2014	[illegible]	Chimiothérapie	2	mixte	cancer	HDJ	OUI	antalgiques	5-10min
4	OUI	OUI	NC	OUI	08/04/2015	HOMME	15/08/1950	urologique	999	01/07/2005	Métastatique	Chimiothérapie	3	mixte	cancer	hospitalisation	oui	antalgiques	5-10min
5	OUI	NON	OUI	NON	08/04/2015	FEMME	15/04/1952	sein	999	15/12/2014	Métastatique	Chimiothérapie	3	mixte	cancer	hospitalisation	oui	antalgiques	15-30min
6	NON	OUI	non	NON	16/04/2015	FEMME	15/08/1950	gastro-intestinal	999	01/04/2009	Métastatique	Chimiothérapie	2	mixte	cancer	hospitalisation	OUI	antalgiques	15-30min
7	non	OUI	oui	non	20/04/2015	FEMME	15/09/1967	sein	999	29/06/2005	Métastatique	Chimiothérapie	3	mixte	cancer	hospitalisation	OUI	antalgiques	15-30min
8	OUI	NON	NON	OUI	23/04/2015	FEMME	15/04/1944	poumon	999	29/06/2005	Métastatique	Chimiothérapie	2	mixte	cancer	hospitalisation	OUI	antalgiques	15-30min
9	OUI	NON	OUI	NON	28/04/2015	FEMME	15/08/1971	sein	999	30/06/2005	Métastatique	Chimiothérapie	3	mixte	les deux	hospitalisation	oui	cortico	<5min
10	oui	non	oui	non	29/04/2015	HOMME	15/01/1946	ORL	999	01/01/2014	Recidive	Chimiothérapie	2	nociceptive	cancer	hospitalisation	OUI	antalgiques	30-60min
11	OUI	NON	OUI	NON	29/04/2015	FEMME	15/10/1968	gastro-intestinal	999	01/05/2014	Métastatique	Chimiothérapie	3	neuropathique	cancer	hospitalisation	OUI	antalgiques	30-60min
12	OUI	OUI	non	OUI	12/05/2015	HOMME	15/02/1953	dermatologique	999	01/06/2012	Métastatique	Chimiothérapie	2	nociceptive	cancer	hospitalisation	OUI	antalgiques	<5MIN
13	NON	OUI	OUI	NON	18/05/2015	HOMME	15/05/1947	urologique	999	15/06/2014	Métastatique	NC	3	mixte	cancer	hospitalisation	OUI	antalgiques	30-60min
14	OUI	NON	non	NON	20/05/2015	HOMME	15/06/1953	dermatologique	999	11/03/2013	Métastatique	Chimiothérapie	3	mixte	cancer	hospitalisation	OUI	antalgiques	30-60min
15	NON	OUI	OUI	non	21/05/2015	HOMME	15/01/1947	urologique	999	01/07/2005	Métastatique	Chimiothérapie	3	nociceptive	cancer	hospitalisation	OUI	antalgiques	>60min
16	OUI	NON	OUI	NON	26/05/2015	HOMME	15/02/1957	dermatologique	999	30/06/2005	Métastatique	Chimiothérapie	3	mixte	cancer	hospitalisation	OUI	position	>60min
17	OUI	NON	OUI	NON	18/06/2015	HOMME	15/04/1937	urologique	999	11/02/2015	Métastatique	chirurgie	2	nociceptive	cancer	hospitalisation	OUI	antalgiques	30-60min
18	OUI	NON	non	OUI	15/06/2015	HOMME	15/06/1962	urologique	999	01/02/2015	Métastatique	Chimiothérapie	3	mixte	les deux	hospitalisation	OUI	glace	15-30min
19	OUI	NON	non	OUI	23/06/2016	FEMME	15/06/1933	gastro-intestinal	999	01/02/2014	Métastatique	Chimiothérapie	4	mixte	cancer	hospitalisation	OUI	antalgiques	>60min
20	OUI	NON	non	OUI	08/07/2015	FEMME	15/06/1963	sein	999	01/03/2014	Métastatique	Chimiothérapie	2	mixte	cancer	hospitalisation	OUI	antalgiques	15-30min
21	OUI	NON	OUI	NON	18/07/2015	HOMME	15/07/1926	urologique	999	03/07/2005	Métastatique	Chimiothérapie	2	nociceptive	cancer	hospitalisation	OUI	antalgiques	30-60min
22	OUI	NON	OUI	NON	23/07/2015	HOMME	15/08/1962	dermatologique	999	01/11/2011	Métastatique	Chimiothérapie	2	mixte	cancer	hospitalisation	OUI	antalgiques	<5min
23	NON	OUI	non	OUI	23/07/2015	FEMME	15/11/1955	autre	999	06/07/2014	[illegible]	Chimiothérapie	2	neuropathique	cancer	hospitalisation	Non	999	<5min
24	OUI	NON	non	OUI	05/08/2015	FEMME	15/04/1942	gynécologique		01/07/2014	Métastatique	Chimiothérapie	3	mixte	cancer	hospitalisation	OUI	antalgiques	15-30min
25	OUI	NON	non	NON	21/08/2015	FEMME	15/09/1939	dermatologique	999	01/06/2006	Métastatique	Chimiothérapie	3	mixte	cancer	hospitalisation	OUI	[illegible]	<5min
26	OUI	NON	non	OUI	28/08/2015	FEMME	15/10/1972	gastro-intestinal	999	01/01/2015	[illegible]	Chimiothérapie	2	mixte	les deux	hospitalisation	OUI	antalgiques	30-60min
27	OUI	NON	non	OUI	01/10/2015	FEMME	15/02/1944	sein		01/11/2012	Métastatique	radiothérapie	2	nociceptive	les deux	hospitalisation	OUI	antalgiques	5-15min
28	OUI	NON	OUI	NON	05/10/2015	FEMME	15/03/1957	gynécologique	999	01/06/2004	Métastatique	Chimiothérapie	3	mixte	cancer	hospitalisation	OUI	antalgiques	30-60min
29	NON	OUI	non	OUI	26/10/2015	HOMME	15/06/1938	gastro-intestinal	999	01/05/2015	Métastatique	Chimiothérapie	3	nociceptive	cancer	hospitalisation	oui	antalgiques	15-30min
30	OUI	NON	OUI	NON	29/10/2015	HOMME	15/04/1975	gastro-intestinal	999	19/06/2015	[illegible]	Chimiothérapie	0	nociceptive	cancer	hospitalisation	OUI	antalgiques	15-30min
31	OUI	NON	non	OUI	12/11/2015	FEMME	15/06/1971	gynécologique	999	29/06/2013	Métastatique	Chimiothérapie	2	nociceptive	cancer	hospitalisation	OUI	antalgiques	NC
32	OUI	NON	OUI	NON	19/11/2015	HOMME	15/02/1953	urologique	999	01/06/2013	Métastatique	Chimiothérapie	2	mixte	les deux	hospitalisation	OUI	[illegible]	<5min
33	OUI	NON	OUI	NON	26/11/2015	HOMME	15/02/1961	sein	999	30/06/2012	Métastatique	Chimiothérapie	3	mixte	cancer	hospitalisation	OUI	antalgiques	15-30min
34	OUI	NON	OUI	NON	04/12/2015	HOMME	15/01/1948	gastro-intestinal	999	04/07/2005	Métastatique	Chimiothérapie	3	nociceptive	cancer	hospitalisation	OUI	antalgiques	15-30min
35	OUI	NON	non	OUI	08/12/2015	HOMME	15/11/1959	dermatologique	999	20/06/2012	Métastatique	Chimiothérapie	1	nociceptive	cancer	hospitalisation	OUI	antalgiques	>60min
36	OUI	NON	OUI	NON	07/12/2015	HOMME	15/12/1968	dermatologique	999	01/06/2011	Métastatique	Chimiothérapie	0	nociceptive	cancer	hospitalisation	OUI	antalgiques	<5min
37	OUI	NON	OUI	NON	17/12/2015	FEMME	15/06/1949	gastro-intestinal	999	01/06/2014	Métastatique	Chimiothérapie	3	nociceptive	cancer	hospitalisation	OUI	antalgiques	15-30min
38	OUI	NON	non	NON	07/01/2016	FEMME	15/08/1969	dermatologique	999	18/06/2006	Métastatique	Chimiothérapie	1	nociceptive	cancer	hospitalisation	OUI	antalgiques	30-60min
39	OUI	NON	non	OUI	01/02/2016	HOMME	15/07/1946	urologique	999	12/10/2015	Métastatique	Chimiothérapie	2	mixte	cancer	hospitalisation	OUI	antalgiques	15-30min
40	NON	OUI	non	OUI	09/02/2016	HOMME	15/05/1935	dermatologique	999	01/09/2011	Métastatique	Chimiothérapie	2	nociceptive	cancer	hospitalisation	oui	antalgiques	5-15min
41	OUI	oui	non	OUI	17/02/2016	HOMME	15/02/1968	urologique	999	01/11/2014	Métastatique	Chimiothérapie	2	mixte	les deux	hospitalisation	oui	position	5-15min
42	OUI	NON	non	OUI	29/02/2016	FEMME	15/06/1963	sein	999	19/07/2013	Métastatique	Chimiothérapie	3	mixte	cancer	hospitalisation	oui	antalgiques	15-30min
43	OUI	NON	OUI	NON	14/03/2016	HOMME	15/07/1975	autre	[illegible]	01/08/2015	Métastatique	Chimiothérapie	2	nociceptive	les deux	hospitalisation	oui	antalgiques	5-15min
44	OUI	NON	non	OUI	22/06/2016	HOMME	15/08/1958	gastro-intestinal	999	01/03/2016	Métastatique	Chimiothérapie	3	mixte	cancer	hospitalisation	OUI	antalgiques	15-30min
45	OUI	oui	non	oui	29/07/2016	FEMME	15/07/1954	gastro-intestinal	999	01/05/2016	[illegible]	Chimiothérapie	3	nociceptive	cancer	hospitalisation	OUI	antalgiques	5-15min
46	OUI	NON	non	OUI	01/08/2016	HOMME	15/05/1951	urologique	999	15/06/2014	Métastatique	radiothérapie	2	mixte	cancer	hospitalisation	OUI	[illegible]	<5min
47	NON	OUI	OUI	OUI	05/09/2016	FEMME	15/05/1952	gastro-intestinal	999	01/02/2016	[illegible]	Chimiothérapie	3	mixte	cancer	hospitalisation	OUI	antalgiques	5-15min
48	OUI	NON	OUI	NON	05/09/2016	FEMME	15/07/1952	sein	999	01/06/2006	Métastatique	Chimiothérapie	2	nociceptive	cancer	hospitalisation	OUI	[illegible]	<5min

49	OUI	NON	non	OUI	08/09/2016	FEMME	15/08/1958	hématologique	999	14/06/2008	Recidive	chirurgie	1	nociceptive	cancer	consultation	OUI	antalgiques	>60min	[illegible]
50	OUI	NON	non	OUI	15/09/2016	FEMME	15/06/1977	gastro-intestinal	999	01/10/2015	Métastatique	Chimiothérapie	2	nociceptive	cancer	hospitalisation	OUI	antalgiques	5-15min	[illegible]
51	OUI	NON	NON	NON	20/09/2016	FEMME	15/04/1953	urologique	999	01/07/2009	Métastatique	Chimiothérapie	2	nociceptive	cancer	hospitalisation	OUI	[illegible]	5-15min	[illegible]
52	NON	NON	OUI	NON	12/10/2016	HOMME	15/04/1962	autre	primitif inconnu	01/05/2016	Métastatique	Chimiothérapie	2	nociceptive	cancer	hospitalisation	OUI	antalgiques	15-30min	[illegible]
53	NON	OUI	NON	OUI	28/11/2016	FEMME	15/12/1965	gynécologique	999	01/06/2015	[illegible]	Chimiothérapie	2	mixte	cancer	hospitalisation	OUI	antalgiques	30-60min	[illegible]
54	OUI	NON	OUI	NON	06/12/2016	FEMME	15/08/1969	hématologique	999	01/02/2015	Recidive	Chimiothérapie	3	mixte	cancer	hospitalisation	NON	antalgiques	15-30min	[illegible]
55	OUI	NON	NON	OUI	08/12/2016	FEMME	15/06/1939	dermatologique	999	01/10/2012	Métastatique	Chimiothérapie	3	nociceptive	cancer	hospitalisation	OUI	antalgiques	5-15min	[illegible]
56	OUI	NON	OUI	NON	14/12/2016	FEMME	15/06/1949	hématologique	999	29/05/2013	Métastatique	Chimiothérapie	3	mixte	cancer	hospitalisation	OUI	antalgiques	5-15min	[illegible]
57	OUI	NON	oui	non	06/02/2017	FEMME	15/01/1948	sein	999	01/02/2013	Métastatique	Chirurgie	3	mixte	cancer	hospitalisation	OUI	antalgiques	30-60min	[illegible]
58	OUI	NON	OUI	NON	11/01/2017	HOMME	15/09/1934	urologique	999	01/12/2014	Métastatique	Chirurgie	3	mixte	cancer	hospitalisation	OUI	antalgiques	30-60min	[illegible]
59	OUI	NON	OUI	NON	18/01/2017	HOMME	15/04/1946	urologique	999	26/02/2016	Métastatique	Chirurgie	2	nociceptive	les deux	hospitalisation	OUI	antalgiques	5-15min	[illegible]
60	NON	non	oui	non	31/01/2017	FEMME	15/05/1961	sein	999	01/01/2007	Métastatique	Chimiothérapie	3	mixte	cancer	hospitalisation	OUI	antalgiques	5-15min	[illegible]
61	OUI	NON	OUI	NON	09/02/2017	FEMME	15/03/1978	dermatologique	999	19/01/2009	Métastatique	Chimiothérapie	2	mixte	cancer	hospitalisation	NON	nc	15-30min	[illegible]
62	NON	NON	OUI	NON	01/03/2017	FEMME	15/07/1973	gynécologique	999	01/07/2016	Métastatique	Chimiothérapie	2	mixte	cancer	hospitalisation	OUI	antalgiques	15-30min	[illegible]
63	OUI	NON	NON	OUI	30/03/2017	FEMME	15/06/1956	ORL	999	01/12/2014	Recidive	Chimiothérapie	1	mixte	cancer	hospitalisation	OUI	antalgiques	30-60min	[illegible]
64	OUI	NON	NON	OUI	05/04/2017	FEMME	15/09/1955	gynécologique	999	01/04/2012	Métastatique	Chimiothérapie	3	nociceptive	cancer	hospitalisation	OUI	antalgiques	30-60min	[illegible]
65	OUI	non	oui	non	05/04/2017	HOMME	15/11/1941	poumon	999	01/03/2017	Métastatique	Chimiothérapie	2	nociceptive	cancer	hospitalisation	OUI	antalgiques	30-60min	[illegible]
66	oui	non	non	non	10/04/2017	FEMME	15/03/1953	gastro-intestinal	sarcome	01/06/2012	[illegible]	Chimiothérapie	4	nociceptive	cancer	hospitalisation	OUI	antalgiques	30-60min	[illegible]
67	OUI	NON	NON	NON	24/04/2017	FEMME	15/11/1979	gynécologique	999	01/12/2016	Métastatique	Chimiothérapie	2	mixte	cancer	hospitalisation	OUI	antalgiques	5-15min	[illegible]
68	OUI	NON	OUI	NON	15/05/2017	HOMME	15/12/1939	gastro-intestinal	999	01/10/2014	Métastatique	Chimiothérapie	3	mixte	cancer	hospitalisation	OUI	antalgiques	15-30min	[illegible]
69	OUI	OUI	NON	OUI	26/06/2017	HOMME	15/06/1962	gastro-intestinal	999	01/07/2015	Métastatique	Chirurgie	2	mixte	cancer	hospitalisation	OUI	position	30-60min	[illegible]
70	oui	NON	OUI	NON	28/06/2017	HOMME	15/01/1954	gastro-intestinal	999	01/01/2017	Métastatique	Chimiothérapie	3	mixte	cancer	hospitalisation	OUI	position	5-15min	[illegible]
71	nc	nc	nc	nc	nc	nc	nc	nc	nc	nc	nc	nc	nc	nc	nc	nc	nc	nc	nc	[illegible]
72	OUI	OUI	NON	OUI	25/09/2017	HOMME	15/02/1964	gastro-intestinal	999	01/10/2015	Métastatique	Chimiothérapie	2	mixte	cancer	hospitalisation	OUI	froid	30-60min	[illegible]
73	OUI	NON	NON	OUI	18/10/2017	FEMME	15/05/1959	dermatologique	999	01/08/2016	Métastatique	Chimiothérapie	1	mixte	cancer	HDJ	OUI	antalgiques	15-30min	[illegible]
74	OUI	NON	OUI	NON	24/10/2017	HOMME	15/11/1964	dermatologique	999	01/12/2014	Métastatique	Radiothérapie	0	nociceptive	cancer	hospitalisation	OUI	position	5-15min	[illegible]
75	OUI	NON	OUI	NON	01/12/2017	FEMME	15/05/1959	poumon	999	01/10/2016	Métastatique	radiothérapie	1	mixte	cancer	hospitalisation	OUI	antalgiques	15-30min	[illegible]
76	NON	NON	NON	OUI	06/12/2017	HOMME	15/11/1943	urologique	999	01/01/2008	Métastatique	Chimiothérapie	3	nociceptive	cancer	hospitalisation	OUI	position	<5min	[illegible]
77	OUI	NON	OUI	NON	28/11/2017	FEMME	15/10/1947	gynécologique	999	01/12/2016	[illegible]	Radiothérapie	2	mixte	cancer	hospitalisation	OUI	position	>60min	[illegible]
78	NON	OUI	NON	OUI	22/11/2017	FEMME	15/12/1983	gastro-intestinal	999	01/11/2016	Métastatique	Chimiothérapie	3	nociceptive	cancer	hospitalisation	OUI	antalgiques	5-15min	[illegible]
79	OUI	OUI	NON	OUI	16/11/2017	HOMME	15/03/1965	hématologique	999	01/06/2017	[illegible]	Chimiothérapie	2	neuropathique	cancer	hospitalisation	oui	antalgiques	30-60min	[illegible]
80	OUI	NON	OUI	NON	02/01/2018	HOMME	15/11/1964	gastro-intestinal	999	01/11/2016	[illegible]	Chimiothérapie	2	mixte	traitement	hospitalisation	OUI	antalgiques	5-15min	[illegible]
81	OUI	NON	OUI	NON	09/01/2018	HOMME	15/10/1961	gastro-intestinal	999	27/11/2016	Métastatique	Chimiothérapie	2	nociceptive	cancer	hospitalisation	OUI	chaud	15-30min	[illegible]
82	OUI	NON	non	OUI	22/01/2018	FEMME	15/04/1941	gynécologique	999	01/11/2017	[illegible]	NC	2	nociceptive	cancer	hospitalisation	NON	nc	<5min	[illegible]
83	OUI	NON	OUI	NON	16/02/2018	FEMME	15/12/1957	gynécologique	999	01/01/1998	Métastatique	Chirurgie	2	nociceptive	cancer	hospitalisation	OUI	position	<5min	[illegible]
84	OUI	NON	OUI	NON	20/02/2018	HOMME	15/10/1959	poumon	999	01/09/2017	Métastatique	Radiothérapie	2	mixte	cancer	hospitalisation	OUI	antalgiques	<5min	[illegible]
85	OUI	NON	NON	OUI	26/02/2018	FEMME	15/05/1957	gynécologique	999	01/02/2009	Métastatique	Chimiothérapie	1	neuropathique	cancer	hospitalisation	OUI	antalgiques	15-30min	[illegible]
86	OUI	OUI	NON	OUI	01/03/2018	HOMME	15/02/1918	hématologique	999	01/05/2012	Recidive	Chimiothérapie	3	nociceptive	cancer	hospitalisation	OUI	antalgiques	5-15min	[illegible]
87	OUI	OUI	non	OUI	13/03/2018	HOMME	15/07/1934	poumon	999	15/09/2017	[illegible]	Chimiothérapie	3	nociceptive	cancer	consultation	NON		>60min	[illegible]
88	NON	OUI	OUI	NON	31/03/2018	FEMME	15/05/1978	gynécologique	999	17/12/2016	Métastatique	Chimiothérapie	2	mixte	cancer	hospitalisation	OUI	antalgiques	15-30min	[illegible]
89	OUI	NON	NON	OUI	04/04/2018	FEMME	15/10/1956	gastro-intestinal	999	01/01/2018	Métastatique	Chimiothérapie	2	mixte	cancer	hospitalisation	OUI	antalgiques	5-15min	[illegible]
90	oui	non	oui	non	19/04/2018	FEMME	15/12/1972	ORL	999	01/09/2015	Métastatique	Radiothérapie	2	mixte	cancer	hospitalisation	OUI	antalgiques	30-60min	[illegible]
91	OUI	NON	OUI	NON	03/05/2018	HOMME	15/09/1943	urologique	999	13/10/2016	Métastatique	Radiothérapie	2	mixte	cancer	hospitalisation	OUI	antalgiques	5-15min	[illegible]
92	oui	non	non	oui	07/05/2018	HOMME	15/08/1936	urologique	999	14/10/2015	Métastatique	Radiothérapie	2	neuropathique	cancer	hospitalisation	OUI	antalgiques	5-15min	[illegible]
93	OUI	NON	NON	OUI	17/05/2018	FEMME	15/11/1944	dermatologique	999	01/02/2012	Métastatique	Radiothérapie	2	nociceptive	cancer	hospitalisation	OUI	antalgiques	30-60min	[illegible]
94	NON	OUI	NON	OUI	22/05/2018	HOMME	15/03/1948	urologique	999	01/12/2015	Métastatique	Chimiothérapie	2	mixte	cancer	consultation	OUI	antalgiques	<5min	[illegible]
95	oui	OUI	NON	OUI	03/07/2018	HOMME	15/11/1945	poumon	999	01/05/2017	[illegible]	Chimiothérapie	2	mixte	cancer	hospitalisation	OUI	antalgiques	5-15min	[illegible]
96	OUI	NON	NON	OUI	11/07/2018	FEMME	15/08/1963	hématologique	999	01/09/2017	Autre	Chimiothérapie	3	mixte	cancer	hospitalisation	OUI	antalgiques	30-60min	[illegible]
97	OUI	NON	NON	OUI	30/07/2018	HOMME	15/04/1961	gastro-intestinal	999	05/11/2015	Métastatique	Chimiothérapie	2	mixte	les deux	hospitalisation	OUI	antalgiques	5-15min	[illegible]
98	OUI	NON	OUI	NON	13/08/2018	HOMME	15/04/1957	urologique	999	01/01/2010	Métastatique	Chimiothérapie	2	nociceptive	cancer	hospitalisation	OUI	chaud	5-15min	[illegible]

99	OUI	NON	NON	OUI	21/08/2018	HOMME	15/04/1955	urologique		999	10/08/2018	Métastatique	Chimiothérapie	3	mixte	cancer	hospitalisation	OUI	antalgiques	<5min	10	3	0	6	[illegible]	nc	nc	somnolence	0	60	OUI	[illegible]	<1/j	OUI	mobilisation	NON		<5min	10	3	0	0	[illegible]	5	NC	somnolence	0	OUI	NON	OUI	NON	OUI	NON	OUI	NON	[illegible]	[illegible]	<1/j	OUI	[illegible]	OUI	ftm	15-30min	4	3	5	5	[illegible]	3	10-20min		0	nc	nc	OUI	[illegible]	4	2	2	0	0,9	0	5	10	6	2	0
100	OUI	NON	OUI	NON	28/08/2018	HOMME	15/03/1958	dermatologique		999	15/06/2017	Métastatique	Chimiothérapie	3	nociceptive	cancer	hospitalisation	OUI	antalgiques	<5min	7	7	6	9	[illegible]	10	10-20min	constipation	0	140	OUI	MI	1-2/j	OUI	mobilisation	OUI	ftm	<5min	7	6	8	10	[illegible]	10	10-20min	absence	0	OUI	non	oui	non	OUI	NON	OUI	NON	[illegible]	MI	1-2/j	OUI	[illegible]	OUI	ftm	<5min	7	6	10	10	[illegible]	10	10-20min	[illegible]	0	120	OUI	OUI	MI	7	0	3	3	0,8	2	5	5	2	2	0
101	OUI	NON	NON	OUI	29/08/2018	HOMME	15/02/1940	ORL		999	15/06/2016	[illegible]	Chimiothérapie	3	mixte	cancer	hospitalisation	OUI	antalgiques	30-60min	10	7	4	0	[illegible]	5	>30min	absence	0	120	OUI	NC	>4/j	NON	999	OUI	ftm	15-30min	10	7	0	3	[illegible]	4	20-30min	absence	0	OUI	NON	NON	OUI	OUI	NON	NON	OUI	[illegible]	face/cou	>4/j	NON	999	OUI	ftm	30-60min	9	7	2	4	[illegible]	8	10-20min	absence	0	nc	nc	nc	nc	nc	nc	nc	nc	nc	nc	nc	nc	nc	nc	nc
102	OUI	NON	NON	NON	29/08/2018	FEMME	15/12/1962	gastro-intestinal		999	15/11/2017	Métastatique	Chimiothérapie	3	mixte	cancer	hospitalisation	OUI	antalgiques	30-60min	8	6	8	10	[illegible]	6	10-20min	absence	0	200		NC	3-4/j	OUI	mobilisation	OUI	ftm	30-60min	10	10	8	8	[illegible]	10	10-20min	absence	0	OUI	NON	NON	OUI	OUI	NON	NON	OUI	[illegible]	[illegible]	3-4/j	NON	999	OUI	[illegible]	30-60min	9	5	10	10	[illegible]	8	20-30min	[illegible]	10	nc	nc	OUI	NC	10	0	8	0	0,5	10	10	10	10	10	10
103	non	non	oui	non	22/11/2018	HOMME	15/03/1950	ORL		999	15/11/2017	[illegible]	Chimiothérapie	2	mixte	cancer	hospitalisation	OUI	antalgiques	>60min	7	4	3	9	[illegible]	6	10-20min	somnolence	5	180	oui	face/cou	3-4/j	OUI	parole	oui	repos	5-15min	8	6	7	9	[illegible]	5	20-30min	absence	0	OUI	NON	OUI	NON	OUI	NON	OUI	NON	[illegible]	face/cou	1-2/j	OUI	parole	OUI	ftm	30-60min	6	4	4	8	[illegible]	6	20-30min	[illegible]	6	200	oui	OUI	face/cou	6	2	3	3	0,8	3	3	5	4	4	5
104	OUI	NON	OUI	NON	19/12/2018	HOMME	15/07/1946	ORL		999	15/06/2016	Métastatique	Chimiothérapie	4	neuropathique	cancer	hospitalisation	OUI	[illegible]	30-60min	10	10	6	10	pallier 2	9	20-30min	absence	0	30	NON	face/cou	>4/j	OUI	mobilisation	OUI	antalgiques	<5min	10	6	10	10	paracetamol	6	10-20min	absence	0	OUI	NON	OUI	NON	OUI	NON	OUI	NON	[illegible]	face/cou	1-2/j	OUI	[illegible]	OUI	[illegible]	<5min	10	10	3	10	pallier 2	10	0-10min	absence	0	30	non	OUI	face/cou	10	2	10	0	1	5	10	10	0	0	10
105	NON	NON	OUI	NON	23/01/2018	FEMME	15/07/1971	gastro-intestinal		999	15/11/2015	Métastatique	Radiothérapie	4	mixte	cancer	hospitalisation	OUI	massages	>60min	8	8	8	10	[illegible]	3	10-20min	somnolence	5	160	OUI	MI	3-4/j	OUI	mobilisation	OUI	ftm	>60min	9	6	7	8	[illegible]	5	10-20min	somnolence	0	OUI	NON	OUI	NON	NON	NON	NON	OUI	[illegible]	MI	3-4/j	OUI	[illegible]	NON	999	15-30min	10	8	7	7	[illegible]	7	0-10min	absence	0	160	OUI	OUI	MI	10	3	4	4	0,5	6	10	10	2	6	0
106	OUI	NON	NON	OUI	17/01/2019	FEMME	15/05/1937	sein	[illegible]		15/01/2014	Métastatique	Chirurgie	3	nociceptive	cancer	hospitalisation	OUI	position	<5min	10	5	10	10	paracetamol	2	aucun effet	absence	0	45	non	dos	3-4/j	OUI	mobilisation	OUI	repos	<5min	10	8	9	10	morphiniques	3	aucun effet	absence	0	OUI	NON	OUI	NON	OUI	NON	OUI	NON	[illegible]	dos	>4/j	OUI	[illegible]	OUI	ftm	<5min	7	5	8	7	[illegible]	10	0-10min	[illegible]	7	90	OUI	OUI	dos	8	3	0	1	0,9	7	8	9	9	5	5
107	OUI	NON	OUI	NON	17/01/2019	FEMME	15/03/1948	hématologique		999	17/07/2012	Métastatique	Chimiothérapie	1	nociceptive	cancer	hospitalisation	OUI	antalgiques	30-60min	9	6	6	5	[illegible]	1	aucun effet	somnolence	5	60	non	dos	>4/j	OUI	mobilisation	OUI	repos	15-30min	6	4	7	6	morphiniques	2	0-10min	somnolence	6	OUI	NON	OUI	NON	OUI	NON	OUI	NON	[illegible]	dos	3-4/j	OUI	transit	OUI	repos	5-15min	5	4	3	3	[illegible]	3	10-20min	[illegible]	3	60	NON	OUI	dos		2	3	2	nc	1	4	4	2	1	2
108	OUI	NON	OUI	NON	11/02/2019	HOMME	15/03/1960	autre	inconnu		05/06/2018	Métastatique	Chimiothérapie	2	mixte	cancer	hospitalisation	OUI	ftm	30-60min	9	6	0	8	[illegible]	5	>30min	absence	0	60	OUI	MS	1-2/j	NC	999	OUI	ftm	30-60min	10	8	0	10	[illegible]	5	20-30min	absence	0	OUI	NON	OUI	NON	OUI	NON	OUI	NON	[illegible]	MS	1-2/j	NON	999	OUI	ftm	30-60min	9	7	5	2	[illegible]	5	10-20min	absence	0	90	oui	OUI	nc	nc	nc	nc	nc	nc	nc	nc	nc	nc	nc	nc
109	OUI	NON	OUI	NON	20/02/2019	HOMME	15/01/1934	urologique		999	12/01/2012	Métastatique	[illegible]	3	mixte	cancer	hospitalisation	OUI	antalgiques	30-60min	9	8	9	10	[illegible]	8	>30min	autre	8	60	OUI	dos	>4/j	OUI	mobilisation	OUI	antalgiques	>60min	8	7	8	10	morphiniques	7	>30min	absence	0	OUI	NON	OUI	NON	OUI	NON	NON	OUI	[illegible]	dos	3-4/j	OUI	[illegible]	OUI	repos	30-60min	9	8	8	10	NC	8	>30min	absence	0	60	OUI	OUI	dos	9	NC	nc	nc	nc	nc	nc	nc	nc	nc	nc
110	OUI	NON	OUI	NON	21/02/2019	FEMME	13/08/1953	sein		999	15/06/2003	Métastatique	Chimiothérapie	2	mixte	cancer	hospitalisation	OUI	antalgiques	15-30min	10	5	5	10	[illegible]	8	20-30min	[illegible]	0	200	OUI	[illegible]	>4/j	OUI	mobilisation	OUI	antalgiques	>60min	10	10	0	10	morphiniques	7	20-30min	[illegible]	3	OUI	NON	OUI	NON	OUI	NON	OUI	NON	[illegible]	MI	>4/j	OUI	[illegible]	OUI	repos	<5min	10	7	4	10	[illegible]	6	0-10min	[illegible]	0	nc	nc	OUI	[illegible]	10	7	5	5	0,8	10	10	10	10	10	10
111	OUI	OUI	NON	OUI	21/02/2019	FEMME	15/11/1954	urologique		999	30/06/2016	Métastatique	Chimiothérapie	1	nociceptive	cancer	hospitalisation	OUI	repos	>60min	10	9	10	10	pallier 2	0	aucun effet	[illegible]	9	60	OUI	MI	1-2/j	OUI	mobilisation	OUI	antalgiques	>60min	9	5	5	5	morphiniques	10	>30min	[illegible]	9	OUI	NON	OUI	NON	OUI	NON	OUI	OUI	[illegible]	MI	>4/j	OUI	[illegible]	OUI	repos	>60min	10	8	4	6	[illegible]	7	>30min	[illegible]	3	90	OUI	OUI	MI	10	2	3	3	0,8	7	10	8	6	6	8
112	OUI	NON	NON	OUI	22/02/2019	HOMME	15/11/1945	urologique		999	15/06/2015	Métastatique	Chimiothérapie	3	nociceptive	cancer	hospitalisation	NON		>60min	10	9	8	10	[illegible]	3	>30min	constipation	5	60	OUI	NC	>4/j	OUI	mobilisation	OUI	ftm	30-60min	10	7	9	10	[illegible]	6	10-20min	somnolence	3	OUI	NON	OUI	NON	OUI	NON	OUI	NON	[illegible]	MI	>4/j	NON	999	NC	999	>60min	10	nc	8	8	[illegible]	nc	nc	nc	nc	60	OUI	OUI	MI	6	0	4	4	0,5	10	10	10	10	10	10
113	NON	OUI	OUI	NON	27/02/2019	FEMME	15/08/1973	dermatologique		999	15/06/2006	Métastatique	[illegible]	1	nociceptive	cancer	consultation	OUI	repos	<5min	9	9	6	10	pallier 2	10	0-10min	absence	0	30	NON	MI	>4/j	OUI	mobilisation	OUI	antalgiques	15-30min	9	9	5	5	morphiniques	10	10-20min	constipation	0	OUI	NON	OUI	NON	OUI	NON	OUI	NON	[illegible]	MI	3-4/j	OUI	mobilisation		[illegible]	15-30min	9	9	3	3	[illegible]	10	10-20min	[illegible]	3	40	NON	OUI	nc	7	0	3	3	0,8	3	6	5	3	7	0
114	OUI	OUI	NON	OUI	12/03/2019	FEMME	15/03/1956	gastro-intestinal		999	15/08/2017	Métastatique	Chimiothérapie	3	mixte	cancer	hospitalisation	OUI	antalgiques	<5min	8	5	4	4	[illegible]	5	10-20min	absence	0	60	NON	NC	1-2/j	OUI	mobilisation	OUI	antalgiques	<5min	6	6	3	1	morphiniques	4	0-10min	absence	0	OUI	NON	OUI	NON	OUI	NON	OUI	NON	[illegible]	MI	1-2/j	OUI	[illegible]	OUI	repos	<5min	5	3	0	0	[illegible]	6	10-20min	absence	0	60	non	OUI	MI	5	2	3	2	0,5	2	0	1	0	2	1
115	OUI	NON	NON	OUI	11/04/2019	HOMME	15/08/1962	urologique		999	15/06/2010	Métastatique	Chimiothérapie	2	mixte	cancer	hospitalisation	OUI	antalgiques	30-60min	10	5	5	9	[illegible]	8	>30min	absence	0	160	OUI	MI	>4/j	NON	999	OUI	antalgiques	30-60min	10	4	5	2	morphiniques	6	>30min	absence	0	OUI	OUI	OUI	OUI	NON	OUI	OUI	NON	[illegible]	MI	>4/j	OUI	[illegible]	OUI	repos	>60min	9	4	6	5	[illegible]	8	>30min	absence	0	200	NON	OUI	MI	7	0	3	2	0,6	5	6	8	3	8	4
116	OUI	NON	OUI	NON	15/04/2019	HOMME	15/11/1954	poumon		999	15/04/2017	[illegible]	Chimiothérapie	2	mixte	cancer	hospitalisation	OUI	chaud	30-60min	9	7	3	8	[illegible]	8	20-30min	absence	0	60	OUI	thorax	1-2/j	NON	999	OUI	antalgiques	30-60min	9	4	5	6	[illegible]	4	20-30min	autre	5	NON	OUI	OUI	NON	NON	OUI	OUI	NON	[illegible]	thorax	3-4/j	NON	999	OUI	repos	30-60min	9	6	8	9	[illegible]	8	10-20min	absence	0	60	OUI	NON	thorax	9	2	2	9	0,5	6	5	nc	8	6	6
117	OUI	NON	OUI	NON	16/04/2019	FEMME	15/05/1958	gastro-intestinal		999	15/04/2018	Métastatique	Chimiothérapie	3	mixte	cancer	hospitalisation	OUI	ftm	5-15min	10	8	10	8	[illegible]	6	10-20min	absence	0	200	OUI	[illegible]	3-4/j	NON	999	OUI	froid	15-30min	10	5	8	8	[illegible]	6	10-20min	absence	0	NC	NC	NC	NC	NC	NC	NC	NC	NC	NC	NC	NC	999	NC	999	NC	nc	nc	nc	nc	NC	nc	nc	nc	nc	nc	nc	NC	nc	nc	nc	nc	nc	nc	nc	nc	nc	nc	nc	nc
118	OUI	NON	OUI	NON	13/05/2019	HOMME	15/12/1964	urologique		999	15/01/2017	Métastatique	radiothérapie	1	nociceptive	cancer	hospitalisation	OUI	ftm	30-60min	9	5	10	10	[illegible]	8	10-20min	constipation	5	480	OUI	[illegible]	3-4/j	NON	999	OUI	ftm	15-30min	9	6	5	6	[illegible]	4	10-20min	absence	0	OUI	NON	OUI	NON	NON	OUI	OUI	NON	[illegible]	[illegible]	<1/j	NON	999	OUI	ftm	5-15min	0	0	0	0	[illegible]	10	10-20min	absence	0	480	OUI	OUI	[illegible]	6	1	5	3	0,7	3	7	8	5	4	8
119	OUI	NON	NON	OUI	15/05/2019	FEMME	15/06/1947	sein		999	15/06/2017	Métastatique	Chimiothérapie	3	mixte	cancer	hospitalisation	OUI	repos	5-15min	5	5	10	10	[illegible]	3	>30min	somnolence	8	180	OUI	thorax	3-4/j	OUI	mobilisation	OUI	ftm	5-15min	8	4	10	10	[illegible]	5	20-30min	somnolence	3	OUI	NON	NON	OUI	OUI	NON	OUI	NON	[illegible]	[illegible]	3-4/j	OUI	[illegible]	OUI	ftm	5-15min	10	5	2	8	[illegible]	8	0-10min	[illegible]	8	180	OUI	OUI	thorax	10	4	5	3	0,5	3	7	NC	0	0	7
120	OUI	NON	OUI	NON	27/06/2019	homme	15/09/1972	gastro-intestinal		999	15/06/2016	Métastatique	Chimiothérapie	2	mixte	cancer	hospitalisation	OUI	ftm	>60min	10	8	8	10	[illegible]	10	10-20min	somnolence	5	120	OUI	[illegible]	3-4/j	OUI	defecation	OUI	ftm	30-60min	10	8	6	10	[illegible]	10	10-20min	somnolence	4	OUI	NON	OUI	NON	NON	OUI	OUI	NON	[illegible]	[illegible]	3-4/j	OUI	[illegible]	OUI	ftm	30-60min	10	10	6	10	[illegible]	8	10-20min	absence	0	120	OUI	OUI	MS	nc	2	3	6	0,6	5	0	nc	1	5	5
121	OUI	NON	OUI	NON	08/07/2019	homme	15/10/1949	gastro-intestinal		999	15/06/2011	metastatique	radiothérapie	3	mixte	cancer	hospitalisation	OUI	ftm	5-15min	8	6	0	9	[illegible]	7	10-20min	absence	0	120	OUI	MS	3-4/j	oui	mobilisation	OUI	ftm	15-30min	7	6	7	3	[illegible]	6	NC	absence	NC	NON	OUI	NON	OUI	NON	NON	NON	OUI	[illegible]	MS	>4/j	NON	999	NON	999	30-60min	10	5	10	5	[illegible]	6	NC	autre	0	90	OUI	OUI	MS	nc	2	3	6	0,6	5	0	NC	1	5	5
122	OUI	NON	NON	OUI	05/08/2019	FEMME	15/10/1965	gastro-intestinal		999	15/10/2015	metastatique	Chimiothérapie	3	mixte	cancer	hospitalisation	OUI	antalgiques	30-60min	10	7	0	10	[illegible]	5	10-20min	absence	0	120	OUI	[illegible]	>4/j	OUI	mobilisation	OUI	antalgiques	>60min	10	8	0	10	morphiniques	7	20-30min	absence	0	NC	NC	NC	NC	NC	NC	NC	NC	NC	NC	NC	NC	999	NC		NC	NC	NC	NC	NC	NC	NC	NC	NC	NC	nc	nc	NC	NC	NC	NC	NC	NC	NC	NC	NC	NC	NC	NC	NC
123	OUI	NON	OUI	NON	05/08/2019	FEMME	15/04/1941	gastro-intestinal		999	15/07/2017	metastatique	Chimiothérapie	2	mixte	cancer	hospitalisation	OUI	antalgiques	30-60min	8	6	4	6	[illegible]	6	20-30min	absence	0	100	OUI	NC	1-2/j	OUI	mobilisation	OUI	antalgiques	30-60min	8	5	0	5	morphiniques	6	>30min	absence	0	NON	NON	OUI	NON	OUI	NON	OUI	NON	[illegible]	[illegible]	1-2/j	OUI	[illegible]	OUI	[illegible]	15-30min	8	6	5	10	[illegible]	5	NC	[illegible]	5	100	OUI	OUI	[illegible]	8	4	5	5	0,4	7	5	5	5	4	5
124	OUI	NON	OUI	NON	21/08/2019	FEMME	15/01/1954	gynécologique		999	15/12/2015	metastatique	Chimiothérapie	3	nociceptive	cancer	hospitalisation	OUI	ftm	5-15min	6	4	6	8	[illegible]	8	10-20min	constipation	3	180	OUI	NC	3-4/j	OUI	mobilisation	OUI	antalgiques	15-30min	7	4	6	5	morphiniques	5	20-30min	somnolence	6	NON	OUI	OUI	NON	NC	NC	NC	NC	NC	NC		NC	999	NC	999	NC	NC	NC	NC	NC	NC	NC	NC	NC	NC	NC	NC	NC	NC	NC	NC	NC	NC	NC	NC	NC	NC	NC	NC	NC
125	OUI	NON	OUI	NON	21/08/2019	HOMME	15/01/1954	urologique		999	15/06/2016	metastatique	Chimiothérapie	3	mixte	cancer	hospitalisation	OUI	ftm	30-60min	10	6	5	3	[illegible]	4	10-20min	absence	0	200	OUI	[illegible]	3-4/j	OUI	mobilisation	OUI	antalgiques	15-30min	9	6	7	7	morphiniques	6	10-20min	absence	0	OUI	NON	OUI	NON	OUI	NON	OUI	NON	[illegible]	[illegible]	1-2/j	OUI	[illegible]	OUI	[illegible]	15-30min	7	5	5	4	[illegible]	6	10-20min	[illegible]	7	120	OUI	OUI	[illegible]	6	3	5	3	0,6	0	6	NC	0	0	0
126	OUI	NON	OUI	NON	22/08/2019	FEMME	15/03/1980	gastro-intestinal		999	15/06/2015	metastatique	Chimiothérapie	2	mixte	cancer	hospitalisation	OUI	repos	>60min	10	5	10	10	pallier 2	5	20-30min	[illegible]	3	30	NON	[illegible]	3-4/j	OUI	mobilisation	OUI	antalgiques	>60min	10	7	10	10	pallier 2	5	20-30min	somnolence	4	OUI	NON	OUI	NON	OUI	NON	OUI	NON	[illegible]	[illegible]	1-2/j	OUI	[illegible]	OUI	[illegible]	15-30min	7	5	2	8	pallier 2	10	20-30min	absence	0	30	NON	OUI	[illegible]	7	0	5	3	0,9	2	7	5	2	1	1
127	OUI	NON	OUI	NON	22/08/2019	HOMME	15/06/1919	urologique		999	15/06/2013	metastatique	Chimiothérapie	3	mixte	cancer	hospitalisation	OUI	antalgiques	30-60min	8	7	5	7	[illegible]	10	10-20min	[illegible]	5	120	OUI	MI	>4/j	OUI	mobilisation	OUI	ftm	30-60min	9	7	4	9	[illegible]	6	20-30min	absence	0	OUI	NON	NON	NON	OUI	NON	OUI	NON	[illegible]	MI	>4/j	OUI	[illegible]	OUI	[illegible]	30-60min	8	8	3	6	[illegible]	8	20-30min	absence	0	120	OUI	OUI	[illegible]	7	2	3	2	0,8	4	10	nc	3	6	0
128	OUI	NON	OUI	NON	29/08/2019	HOMME	15/08/1956	poumon		999	15/06/2016	metastatique	Chimiothérapie	2	mixte	cancer	hospitalisation	OUI	antalgiques	>60min	9	5	3	10	[illegible]	0	aucun effet	constipation	8	960	OUI	thorax	3-4/j	OUI	mobilisation	OUI	antalgiques	>60min	8	5	3	10	morphiniques	0	aucun effet	constipation	6	OUI	NON	NON	OUI	OUI	NON	NON	OUI	[illegible]	[illegible]	1-2/j	NON	999	OUI	[illegible]	30-60min	10	6	4	4	[illegible]	5	20-30min	[illegible]	5	960	OUI	OUI	thorax	10	0	3	3	0,3	0	10	10	0	2	8
129	OUI	NON	OUI	NON	30/08/2019	homme	15/05/1964	poumon		999	15/08/2018	metastatique	Chimiothérapie	3	mixte	cancer	hospitalisation	OUI	antalgiques	15-30min	9	7	6	7	[illegible]	8	20-30min	somnolence	4	60	NON	MI	3-4/j	OUI	mobilisation	OUI	antalgiques	30-60min	7	5	4	7	morphiniques	8	20-30min	constipation	7	NC	NC	NC	NC	NC	NC	NC	NC	NC	NC	NC	NC	999	NC	999	NC	NC	NC	NC	NC	NC	NC	NC	NC	NC	nc	NC	NC	NC	NC	NC	NC	NC	NC	NC	NC	NC	NC	NC	NC
130	NON	OUI	NON	OUI	05/09/2019	HOMME	15/12/1939	urologique		999	15/03/2019	metastatique	radiothérapie	2	mixte	cancer	hospitalisation	OUI	antalgiques	<5min	10	10	0	10	[illegible]	3	NC	constipation	3	60	NON	NC	NC	NC	999	NC	999	NC	NC	NC	NC	NC	NC	NC	NC	NC	NC	NC	NC	NC	NC	NC	NC	NC	NC	NC	NC	NC	NC	999	NC	999	NC	NC	NC	NC	NC	NC	NC	NC	NC	NC	nc	nc	NC	NC	NC	NC	NC	NC	NC	NC	NC	NC	NC	NC	NC
131	OUI	NON	OUI	NON	03/09/2019	HOMME	15/11/1969	urologique		999	15/02/2016	metastatique	radiothérapie	3	mixte	cancer	hospitalisation	OUI	antalgiques	<5min	10	7	6	6	[illegible]	8	0-10min	somnolence	0	150	OUI	MS	3-4/j	OUI	mobilisation	OUI	ftm	5-15min	9	7	7	9	[illegible]	10	0-10min	somnolence	0	OUI	NON	OUI	NON	OUI	NON	OUI	NON	[illegible]	MS	1-2/j	OUI	[illegible]	OUI	ftm	5-15min	7	9	8	8	[illegible]	9	20-30min	[illegible]	7	150	OUI	OUI	MS	9	2	6	4	0,7	9	6	6	9	1	8
